# Low gait speed is better than frailty and sarcopenia at identifying the risk of disability in older adults

**DOI:** 10.1093/ageing/afaf104

**Published:** 2025-04-23

**Authors:** Aline Fernanda de Souza, Dayane Capra de Oliveira, Paula Camila Ramírez, Roberta de Oliveira Máximo, Mariane Marques Luiz, Maicon Luís Bicigo Delinocente, Andrew Steptoe, Cesar de Oliveira, Tiago da Silva Alexandre

**Affiliations:** Department of Physical Therapy, Postgraduate Program in Physical Therapy, Federal University of Sao Carlos, Sao Carlos, Sao Paulo, Brazil; Department of Physical Therapy, Postgraduate Program in Physical Therapy, Federal University of Sao Carlos, Sao Carlos, Sao Paulo, Brazil; Facultad de Salud, Escuela de Fisioterapia, Universidad Industrial de Santander, Bucaramanga, Colombia; Department of Gerontology, Postgraduate Program in Gerontology, Federal University of Sao Carlos, Sao Carlos, Sao Paulo, Brazil; Department of Physical Therapy, Postgraduate Program in Physical Therapy, Federal University of Sao Carlos, Sao Carlos, Sao Paulo, Brazil; Department of Gerontology, Postgraduate Program in Gerontology, Federal University of Sao Carlos, Sao Carlos, Sao Paulo, Brazil; Department of Behavioural Science and Health, University College London, London, UK; Department of Epidemiology and Public Health, University College London, London, UK; Department of Physical Therapy, Postgraduate Program in Physical Therapy, Federal University of Sao Carlos, Sao Carlos, Sao Paulo, Brazil; Department of Gerontology, Postgraduate Program in Gerontology, Federal University of Sao Carlos, Sao Carlos, Sao Paulo, Brazil; Department of Epidemiology and Public Health, University College London, London, UK; Department of Gerontology, Postgraduate Program in Physical Therapy, Federal University of Sao Carlos, Sao Carlos, Sao Paulo, Brazil

**Keywords:** frail older people, sarcopenic older people, gait speed, slowness, older people

## Abstract

**Objective:**

To compare frailty, sarcopenia and their respective components to determine which is more effective in identifying the risk of disability in basic and instrumental activities of daily living (BADL and IADL, respectively).

**Methods:**

A longitudinal study involving 3,637 individuals without disabilities concerning BADL and 3,696 individuals without disabilities regarding IADL at baseline. Frailty was defined using the phenotype. Sarcopenia was determined according to the criteria proposed by the *EWGSOP2*: low strength (grip strength <27 kg for men and <16 kg for women), low skeletal muscle mass index (<9.36 kg/m^2^ for men and <6.73 kg/m^2^ for women) and low physical performance (gait speed ≤0.8 m/s). In addition to the complete constructs, each component was assessed. Poisson mixed models were utilised, with the outcome identified as incident cases of disability over 8 years, adjusted for covariates. Results: Pre-frailty was associated with a 17% and 18% annual increase in the risk of disability for BADL and IADL, respectively. These figures were 27% and 28% for individuals classified as frail. Sarcopenia was not associated with an increased risk of disability. Amongst the components of frailty and sarcopenia, low physical performance, assessed by gait speed ≤0.8 m/s, was the most effective for identifying the risk of disability (12% per year for BADL and 14% per year for IADL).

**Conclusion:**

In clinical practice, low physical performance (gait speed ≤0.8 m/s) may be the preferred tool for screening the risk of functional decline in older adults.

## Key Points

- A gait speed of ≤0.8 m/s is as effective as frailty in identifying the risk of disability.
- Gait speed is the preferred tool for screening the risk of functional decline in older adults.
- Sarcopenia is not linked to a higher risk of disability.

## Introduction

Functional disability arises from physical, cognitive and emotional impairments [1–3]. Both frailty [4] and sarcopenia [5] are significant risk factors for functional disability. The frailty phenotype is characterised by unintentional weight loss, weakness, slowness, exhaustion and low levels of physical activity [6]. The latest operational definition of sarcopenia proposed by the *European Working Group on Sarcopenia and Older People 2* (*EWGSOP2*) in 2019 integrates low strength and low muscle mass with low physical performance as an aggravating factor [7].

The assessment of both frailty and sarcopenia involves determining gait speed and muscle strength [8], despite differences in the clinical definitions and terminology of the two conditions. In the frailty phenotype, slowness is defined as the 20th percentile of gait speed performance, considering average height and sex, whilst weakness is determined by the 20th percentile of grip strength, accounting for body mass index (BMI) quartiles and sex [6]. In sarcopenia, low physical performance is defined by a gait speed of ≤0.8 m/s [7, 9–11], whereas low strength is defined as grip strength <27 kg for men and <16 kg for women [7].

Few studies have analysed both frailty and sarcopenia, along with their respective components, to determine which is more effective in identifying the risk of disability in clinical practice. For instance, Oliveira *et al.* [12] followed up with over 1,500 British individuals aged 60 years and older without disability at baseline to ascertain which frailty and its components were best at identifying the risk of disability concerning basic activities of daily living (BADL) and instrumental activities of daily living (IADL). The authors found slowness to be a better indicator than frailty for men and women over a 12-year follow-up period [12]. In a study on sarcopenia, Cesari *et al.* [13] tracked 922 Italian individuals aged 65 years and older, also without disability at baseline, to evaluate the predictive power of each component regarding the incidence of disability in BADL over 9 years. They discovered that low physical performance was the only component linked to the outcome in both sexes [13].

Although both constructs are linked to functional disability, their applicability in clinical practice remains below the expectations of geriatricians and specialists in gerontology. It is crucial to explore whether a single component of these constructs would be more effective than the complete construct in identifying the risk of disability in BADL and IADL, aiming to streamline the gerontological assessment process by saving time and reducing costs. Consequently, this study aimed to compare the frailty phenotype and the sarcopenia construct (EWGSOP2), along with their respective components, to determine which most accurately identifies the risk of disability in BADL and IADL for individuals aged 60 years and older over an 8-year follow-up period.

## Methods

The data analysed were from the *English Longitudinal Study of Ageing* (*ELSA*). A previous publication provides further details on *ELSA* [14, 15].

The present study utilised data from Wave 2 of the *ELSA* study (2004) as the baseline, marking the first time that anthropometric measures and physical performance were collected. Amongst the 6,182 participants aged 60 years and older, 1,534 were excluded due to disabilities related to BADL at baseline, and 1,011 were excluded due to insufficient information to define sarcopenia, frailty or covariates, resulting in a final sample of 3,637 participants for the analysis of BADL. Similarly, 1,521 were excluded due to disabilities related to IADL at baseline, and 965 were excluded for lacking information to define sarcopenia, frailty, or covariates, yielding a final sample of 3,696 participants for the analysis of IADL.

### Frailty

Frailty was assessed using the phenotype (2001) [6, 16–20]. Alongside the phenotype construct, each component was analysed individually as a dichotomous variable (present or absent). The supplementary material (*Frailty* section) offers detailed information on the frailty assessment.

### Sarcopenia

Sarcopenia was defined according to the *EWGSOP2* criteria [7, 10, 11, 20–27]. In addition to the overall sarcopenia construct, each component was evaluated separately in the analyses as a dichotomous variable (present or absent). The supplementary material (*Sarcopenia* section) provides detailed information on assessing sarcopenia.

### Basic and instrumental activities of daily living

The basic activities of daily living were examined using the modified Katz index [2], and the instrumental activities of daily living were assessed using the adapted Lawton scale [3]. Only individuals with no difficulties related to any BADL and IADL at baseline were included. The incidence of difficulties with BADL and IADL during the 8-year follow-up period was analysed, with scores ranging from 0 to 6 for each in the subsequent waves of the *ELSA* study. Detailed information on the assessment of basic and instrumental activities of daily living is available in the supplementary material (*Basic and Instrumental activities of daily living* section).

### Covariates

Variables identified in the literature as associated with disability were considered, including age, sex, race, marital status, education level, total household wealth, smoking, alcohol consumption, physical activity level [18–20, 28, 29], self-reported systemic arterial hypertension, diabetes mellitus, cancer, lung disease, heart disease, stroke, osteoarthritis, osteoporosis, instances of falls in the previous year and symptoms of depression, along with results from a memory test and body mass index [16, 17, 30, 31]. Comprehensive details can be found in the supplementary material (*Covariates* section).

### Statistical analyses

The characteristics of the sample at baseline were expressed as mean, standard deviation and proportion. Poisson mixed models were conducted using the XTPOISSON command in Stata17® SE (Stata Corp, College Station, TX, USA) to estimate the risk of disability on BADL and IADL separately. This analytical approach is commonly used to determine the distribution of count data and for outcomes with non-negative whole numbers, with the association measure expressed as relative risk (RR) [32, 33]. Since all participants were free of disability for both BADL and IADL at baseline, the estimates of the mixed models represented the RR in the change of scores on the BADL and IADL scales during the 8-year follow-up (an increase in BADL and IADL scale scores each year).

The final model presented the RR of the incidence of disability on BADL and IADL per year according to the following criteria: (i) frailty phenotype; (ii) sarcopenia construct; (iii) coexistence of frailty phenotype and sarcopenia construct; (iv) each component of the frailty phenotype—unintentional weight loss, exhaustion, weakness, slowness and low physical activity level (yes or no); each component of the sarcopenia construct—low strength, low skeletal muscle mass and low physical performance (yes or no) and (v) coexistence of the components of the frailty phenotype and sarcopenia construct.

A comprehensive modelling approach was employed [34], with adjustments made for a wide range of covariates established *a priori* by the literature as associated with disability. All covariates were treated as time-varying variables (variables that change over time for individuals) [35]. The results were compared using risk ratios (RRs) and their corresponding 95% confidence intervals (CIs). In all analyses, a *P*-value of <0.05 indicated statistical significance. Collinearity amongst variables was assessed using the variance inflation factor, with collinearity considered present when the Variance Inflation Factor (VIF) >10 [36]. However, no collinearity was detected in the analyses. To address survival bias and mitigate the impact of losses to follow-up, inverse probability weighting (IPW) was employed; this method calculates the likelihood of participation and survival of individuals during the follow-up period, incorporating these aspects into the analyses [37–41]. Additionally, comparative analyses were conducted on the baseline characteristics of participants who remained throughout the follow-up period versus those lost to follow-up during the first 4 years or over the 8-year period. Differences between these groups were evaluated using the chi-squared test and Student’s t-test, with a *P*-value of <0.05 regarded as statistically significant.

### Ethical approval and informed consent

The National Research Ethics Service (London Multicentre Research Ethics Committee [MREC/01/2/91]) has approved the *ELSA* study, and all participants provided signed informed consent statements.

## Results

Of the 3,637 participants free of disability in BADL at the start of the study, 74.1% (2,696) and 55.7% (2,026) were reassessed at 4 years and 8 years of follow-up, respectively. Amongst the 3,696 participants free of disability in IADL at the beginning of the study, 75.2% (2,779) and 56.8% (2,100) were reassessed at 4 years and 8 years of follow-up, respectively.

The average age of participants free from disability in both BADL and IADL at baseline was 70 years. Women and individuals with a spouse or partner (32.6% in BADL and 31.7% in IADL), those with 0–11 years of education (54.7% in BADL and 53.9% in IADL), and those wealthier predominated in the sample (24.2% in BADL and 23.9% in IADL). Former smokers (50.6% in BADL and 51.3% in IADL), frequent drinkers (41.1% in BADL and 41.7% in IADL) and individuals who engaged in light physical activity also stood out in the sample (95.6% in BADL and 96.1% in IADL). In terms of health conditions, systemic arterial hypertension was the most prevalent (44.9% in BADL and 45.4% in IADL), followed by osteoarthritis (33.9% in BADL and 34.7% in IADL) and heart disease (22.6% in BADL and 21.9% in IADL). Most participants were classified as overweight (46.1% in BADL and 45.6% in IADL) (Table 1).

**Table 1:** Socioeconomic, behavioural, clinical and anthropometric characteristics of participants in the *ELSA* study (2004) without disabilities in BADL and IADL at baseline.

	BADL n = 3,637	IADL n = 3,696
Socioeconomic characteristics		
Age, (mean ± SD)	(n = 3,637) 69.9 ± 7.2	(n = 3,696) 69.8 ± 7.0
Sex (female), %	(n = 1,970) 54.2	(n = 1,960) 53.0
Race (white), %	(n = 3,581) 98.5	(n = 3,642) 98.5
Marital status (without conjugal life), %	(n = 1,185) 32.6	(n = 1,171) 31.7
Schooling, %		
>13 years	(n = 856) 23.5	(n = 876) 23.7
12–13 years	(n = 793) 21.8	(n = 827) 22.4
0–11 years	(n = 1,988) 54.7	(n = 1,993) 53.9
Total family wealth (quintiles), %		
Fifth quintile (highest)	(n = 881) 24.2	(n = 884) 23.9
Fourth quintile	(n = 803) 22.1	(n = 825) 22.3
Third quintile	(n = 739) 20.3	(n = 780) 21.1
Second quintile	(n = 664) 18.3	(n = 658) 17.8
First quintile (lowest)	(n = 511) 14.0	(n = 506) 13.7
Not declared	(n = 39) 1.1	(n = 43) 1.2
Behavioural characteristics		
Smoking, %		
Non-smoker	(n = 1,375) 37.8	(n = 1,389) 37.6
Former smoker	(n = 1,839) 50.6	(n = 1,897) 51.3
Smoker	(n = 423) 11.6	(n = 410) 11.1
Alcohol intake, %		
Non-drinker or intake up to once per week	(n = 625) 17.2	(n = 618) 16.7
Intake two to six times per week	(n = 1,494) 41.1	(n = 1,542) 41.7
Daily intake	(n = 1,204) 33.1	(n = 1,233) 33.4
Not declared	(n = 314) 8.6	(n = 303) 8.2
Physical activity, %		
Vigorous	(n = 5) 0.1	(n = 5) 0.1
Moderate	(n = 68) 1.9	(n = 69) 1.9
Light	(n = 3,477) 95.6	(n = 3,552) 96.1
Inactive	(n = 87) 2.4	(n = 70) 1.9
Health conditions		
Systemic arterial hypertension, (yes) %	(n = 1,634) 44.9	(n = 1,679) 45.4
Diabetes mellitus (yes), %	(n = 276) 7.6	(n = 289) 7.8
Cancer (yes), %	(n = 331) 9.1	(n = 325) 8.8
Lung disease (yes), %	(n = 604) 16.6	(n = 589) 15.9
Heart disease (yes), %	(n = 822) 22.6	(n = 811) 21.9
Stroke (yes), %	(n = 146) 4.0	(n = 128) 3.5
Osteoarthritis (yes), %	(n = 1,233) 33.9	(n = 1,283) 34.7
Osteoporosis (yes), %	(n = 228) 6.3	(n = 229) 6.2
Falls (yes), %	(n = 987) 27.1	(n = 996) 27.0
Depressive symptoms (yes), %	(n = 371) 10.2	(n = 359) 9.7
Memory score, (mean—SD)	(n = 3,637) 9.7 ± 3.4	(n = 3,696) 9.7 ± 3.3
Anthropometry		
Body mass index, %		
Eutrophic (≥18.5 kg/m^2^ BMI <25 kg/m^2^)	(n = 1,050) 28.9	(n = 1,022) 27.6
Underweight (<18.5 kg/m^2^)	(n = 32) 0.9	(n = 28) 0.8
Overweight (≥25 kg/m^2^ BMI <30 kg/m^2^)	(n = 1,679) 46.1	(n = 1,686) 45.6
Obesity (≥30 kg/m^2^)	(n = 876) 24.1	(n = 960) 26.0

Note: Data is expressed in proportions, as well as means and standard deviations.

Approximately 5% of participants free from disabilities in BADL (191) and IADL (169) were frail, whilst 41% (1,490 in BADL and 1,506 in IADL) were pre-frail. Weakness was the most prevalent component of frailty, followed by exhaustion, slowness, low physical activity levels and unintentional weight loss. In analysing the sarcopenia construct, around 2% (74 in BADL and 67 in IADL) of participants had severe sarcopenia, about 1% (45 in BADL and 41 in IADL) had sarcopenia and approximately 4% (140 in BADL and 147 in IADL) had probable sarcopenia. Despite the low prevalence of probable sarcopenia, sarcopenia and severe sarcopenia, the prevalence of low physical performance (gait speed ≤0.8 m/s) was approximately 28% (1,030 in BADL and 1,014 in IADL) (Table 2).

**Table 2:** Prevalence of frailty, sarcopenia and their respective components amongst *ELSA* study participants (2004) without disabilities in BADL and IADL at baseline.

	BADL n = 3,637	IADL n = 3,696
Frailty		
Non-frail, %	(n = 1,956) 53.8	(n = 2,021) 54.7
Pre-frail, %	(n = 1,490) 41.0	(n = 1,506) 40.7
Frail, %	(n = 191) 5.2	(n = 169) 4.6
Components of frailty		
Unintentional weight loss (yes), %	(n = 32) 0.9	(n = 28) 0.8
Exhaustion (yes), %	(n = 777) 21.4	(n = 771) 20.9
Weakness (yes), %	(n = 855) 23.5	(n = 846) 22.9
Slowness (yes), %	(n = 466) 12.8	(n = 457) 12.4
Low physical activity level (yes), %	(n = 392) 10.8	(n = 334) 9.0
Sarcopenia		
No sarcopenia, %	(n = 3,378) 92.9	(n = 3,441) 93.1
Probable sarcopenia, %	(n = 140) 3.9	(n = 147) 4.0
Sarcopenia, %	(n = 45) 1.2	(n = 41) 1.1
Severe sarcopenia, %	(n = 74) 2.0	(n = 67) 1.8
Components of sarcopenia		
Muscle strength (<27/16 kg), %	(n = 259) 7.1	(n = 255) 6.9
Skeletal muscle mass index (<9.36/6.73 kg/m^2^), %	(n = 902) 24.8	(n = 859) 23.2
Physical performance (≤0.8 m/s), %	(n = 1,030) 28.3	(n = 1,014) 27.4

Note: Data expressed in proportions.

### Basic activities of daily living

In the models where frailty and sarcopenia were analysed as non-coexisting conditions, pre-frail and frail participants had a 17% and 27% increased risk of disability per year, respectively, compared to non-frail participants during the 8-year follow-up period. Similarly, participants with severe sarcopenia faced an 8% higher risk of disability per year compared to non-sarcopenic individuals (Table 3, Supplementary Figure S1). However, in the models that examined the coexistence of frailty and sarcopenia, only pre-frail and frail participants showed an elevated risk of disability compared to non-frail participants (Table 3).

**Table 3:** Estimates of Poisson mixed models as a function of frailty and sarcopenia status for the incidence of disability on BADL (3,637 individuals) and IADL (3,696 individuals) over an 8-year follow-up (2004–2012).

	Frailty	Sarcopenia	Frailty + Sarcopenia
	Relative Risk (95% CI)	Relative Risk (95% CI)	Relative Risk (95% CI)
BADL			
Time, years	0.35 (0.29–0.42)^**^	0.53 (0.47–0.61)^**^	0.33 (0.27–0.41)^**^
Frailty			
Non-frail	Reference		Reference
Pre-frail	1.17 (1.11–1.23)^**^		1.17 (1.11–123)^**^
Frail	1.27 (1.19–1.35)^**^		1.27 (1.19–1.35)^**^
Sarcopenia			
No sarcopenia		Reference	Reference
Probable sarcopenia		1.03 (0.99–1.07)	0.99 (0.95–1.04)
Sarcopenia		1.05 (0.93–1.18)	1.03 (0.90–1.16)
Severe sarcopenia		1.08 (1.01–1.15)^*^	1.01 (0.95–1.08)
IADL			
Time, years	1.01 (0.76–1.35)	1.44 (1.14–1.81)^*^	1.04 (0.78–1.38)
Frailty			
Non-frail	Reference		Reference
Pre-frail	1.18 (1.13–1.24)^**^		1.18 (1.12–1.24)^**^
Frail	1.27 (1.20–1.35)^**^		1.28 (1.20–1.35)^**^
Sarcopenia			
No sarcopenia		Reference	Reference
Probable sarcopenia		1.05 (1.01–1.09)^**^	1.01 (0.97–1.05)
Sarcopenia		1.01 (0.93–1.10)	0.93 (0.85–1.03)
Severe sarcopenia		1.01 (0.95–1.06)	0.97 (0.91–1.03)

Note: CI: confidence intervals; BADL: Basic Activities of Daily Living; IADL: Instrumental Activities of Daily Living; Models adjusted by age, sex, race, marital status, total family wealth, schooling, smoking, alcohol intake, physical activity level, systemic arterial hypertension, diabetes mellitus, cancer, lung disease, heart disease, stroke, osteoarthritis, osteoporosis, falls, depressive symptoms. ^*^P < 0.05; ^**^P < 0.001.

In the analysis focusing solely on the frailty components, a 10% increased risk of disability per year was identified for individuals exhibiting slowness. This rate was 8% per year for those experiencing exhaustion, 7% for individuals with low physical activity levels and 4% for those suffering from weakness (Table 4, Supplementary Figure S2). In the analysis concentrating exclusively on sarcopenia components, a 16% higher risk of disability per year was observed for individuals with low physical performance, whilst this rate was 4% per year for those with low strength (Table 4, Supplementary Figures S1 and S2). However, in the analysis of the components of both constructs, the elevated risk of disability was 12% per year for individuals with low physical performance, 7% per year for those with exhaustion, 6% per year for individuals with low physical activity levels and 4% per year for those with slowness (Table 4).

**Table 4:** Estimates of Poisson mixed models as functions of components of frailty phenotype and sarcopenia for the incidence of disability in BADL amongst 3,637 participants over an 8-year follow-up from 2004 to 2012.

	Frailty	Sarcopenia	Frailty + Sarcopenia
	Relative Risk (95% CI)	Relative Risk (95% CI)	Relative Risk (95% CI)
Time, years	0.41 (0.35–0.49)^**^	0.48 (0.40–0.56)^**^	0.38 (0.31–0.47)^**^
Unintentional weight loss			
No	Reference		Reference
Yes	1.01 (0.98–1.05)		1.01 (0.97–1.05)
Exhaustion			
No	Reference		Reference
Yes	1.08 (1.05–1.13)^**^		1.07 (1.03–1.12)^**^
Weakness			
No	Reference		Reference
Yes	1.04 (1.01–1.08)^*^		1.03 (0.99–1.07)
Slowness			
No	Reference		Reference
Yes	1.10 (1.06–1.14)^**^		1.04 (1.01–1.08)^*^
Low physical activity level			
No	Reference		Reference
Yes	1.07 (1.03–1.11)^**^		1.06 (1.02–1.10)^**^
Muscle strength			
Normal		Reference	Reference
Low		1.04 (1.01–1.08)^*^	1.00 (0.96–1.04)
Skeletal muscle mass index			
≥9.36/6.73 kg/m^2^		Reference	Reference
<9.36/6.73 kg/m^2^		0.98 (0.92–1.05)	0.99 (0.92–1.06)
Physical performance			
>0.8 m/s		Reference	Reference
≤0.8 m/s		1.16 (1.11–1.20)^**^	1.12 (1.07–1.17)^**^

Note: CI: confidence intervals; BADL: Basic Activities of Daily Living; IADL: Instrumental Activities of Daily Living; Models adjusted by age, sex, race, marital status, total family wealth, schooling, smoking, alcohol intake, physical activity level, systemic arterial hypertension, diabetes mellitus, cancer, lung disease, heart disease, stroke, osteoarthritis, osteoporosis, falls, depressive symptoms. ^*^P < 0.05; ^**^P < 0.001.

### Instrumental activities of daily living

In the models where frailty and sarcopenia were analysed as non-coexisting conditions, all pre-frail and frail participants exhibited a higher risk of disability compared to non-frail participants during the 8-year follow-up period. Similarly, participants with probable sarcopenia faced a 5% increased risk of disability each year compared to non-sarcopenic participants (Table 3, Supplementary Figure S1). In the models that considered the coexistence of frailty and sarcopenia, however, it was only the pre-frail and frail participants who showed a greater risk of disability compared to non-frail participants (Table 3).

In the analysis focused solely on frailty components, the risk of disability increased by 9% per year for individuals with a low physical activity level, 8% per year for those experiencing exhaustion and 8% per year for those exhibiting slowness (Table 5, Supplementary Figure S3). In the examination concentrated on the components of sarcopenia, the risk of disability increased by 19% per year for individuals with low physical performance (Table 5, Supplementary Figure S1). However, in the analysis covering components of both constructs, the risk of disability was 14% per year for those with low physical performance, 8% per year for individuals with exhaustion, 7% per year for those with a low physical activity level and 4% per year for those showing slowness (Table 5).

**Table 5:** Estimates of Poisson mixed models as a function of components of frailty phenotype and sarcopenia for the incidence of disability on IADL in 3,696 participants over an 8-year follow-up (2004–2012).

	Frailty	Sarcopenia	Frailty + Sarcopenia
	Relative Risk (95% CI)	Relative Risk (95% CI)	Relative Risk (95% CI)
Time, years	1.10 (0.82–1.47)	1.27 (0.94–1.73)	1.17 (0.86–1.59)
Unintentional weight loss			
No	Reference		Reference
Yes	0.98 (0.95–1.02)		0.98 (0.95–1.02)
Exhaustion			
No	Reference		Reference
Yes	1.08 (1.02–1.12)^**^		1.08 (1.04–1.12)^**^
Weakness			
No	Reference		Reference
Yes	1.03 (0.99–1.07)		1.03 (0.99–1.07)
Slowness			
No	Reference		Reference
Yes	1.08 (1.05–1.12)^**^		1.04 (1.01–1.08)*
Low physical activity level			
No	Reference		Reference
Yes	1.09 (1.05–1.13)^**^		1.07 (1.03–1.11)^**^
Muscle strength			
Normal		Reference	Reference
Low		1.02 (0.99–1.06)	0.98 (0.94–1.02)
Skeletal muscle mass index			
≥9.36/6.73 kg/m^2^		Reference	Reference
<9.36/6.73 kg/m^2^		0.99 (0.94–1.05)	0.99 (0.92–1.05)
Physical performance			
>0.8 m/s		Reference	Reference
≤0.8 m/s		1.19 (1.15–1.24)^**^	1.14 (1.09–1.19)^**^

Note: CI: confidence intervals; BADL: Basic Activities of Daily Living; IADL: Instrumental Activities of Daily Living; Models adjusted by age, sex, race, marital status, total family wealth, schooling, smoking, alcohol intake, physical activity level, systemic arterial hypertension, diabetes mellitus, cancer, lung disease, heart disease, stroke, osteoarthritis, osteoporosis, falls, depressive symptoms. ^*^P < 0.05; ^**^P < 0.001.

The comparative analyses of the baseline characteristics of participants who remained throughout the follow-up period and those lost to follow-up during the first 4 or 8 years revealed that those lost to follow-up were older, had lower levels of education and wealth, smoked more, experienced higher rates of arterial hypertension, cancer, lung disease, heart disease, stroke, malnutrition, frailty and sarcopenia, and exhibited poorer memory performance (*P* < 0.05) compared to those who remained in the study for the entire follow-up period (Supplementary Tables S2 and S3).

## Discussion

In this study, low physical performance, indicated by a gait speed of ≤0.8 m/s, proved to be as effective as the frailty phenotype and more effective than the concept of sarcopenia in identifying the risk of disability in BADL and IADL amongst older adults.

The decline in physical functioning is multifactorial [42–45] and becomes more pronounced with age, yet it does not occur linearly [46]. In a conceptual model, Rivera and colleagues emphasised six domains contributing to the maintenance or decline of physical functioning: the central nervous system, peripheral nervous system, musculoskeletal system, osteoarticular system (bones and joints), perceptual system and energy [45]. Therefore, to preserve activities of daily living, it is essential to maintain these systems throughout life. Sarcopenia, frailty and their respective components are significant conditions that can negatively impact these systems and, as a result, hinder the performance of daily activities.

For instance, frailty was the condition with the most substantial effect size regarding the increased risk of disability for both BADL and IADL. Aguilar-Navarro *et al.* [47] and Makizako *et al.* [48] also identified frailty as a risk factor for the incidence of disability [47, 48]. This can be explained by the fact that the frailty phenotype includes nearly all the domains proposed by Rivera that contribute to the maintenance or decline of physical functioning. The musculoskeletal system is reflected in its components weakness and slowness. The osteoarticular system can influence the slowness component. The perceptual system and energy relate to the exhaustion component; together, these three systems can affect the low physical activity level component. Therefore, regardless of the combination of components that leads to pre-frailty or frailty, it remains a risk factor for disability [6, 45], as demonstrated in this investigation.

This study advances by comparing the weight of each frailty component in relation to the risk of disability, pre-frailty, frailty, sarcopenia and the components of sarcopenia, acknowledging that the unique combination of these components differs amongst individuals. This comparison shows that low physical performance, characterised by a gait speed of ≤0.8 m/s- one of the components of sarcopenia is as effective as frailty in identifying the risk of disability.

Oliveira *et al.* [12] and Provencher *et al.* [49] found similar results, demonstrating that the slowness component alone was as effective as the complete frailty phenotype in identifying poorer trajectories in the incidence of disability in BADL and IADL [12, 49]. In our study, however, we compared slowness defined by Fried’s phenotype to low physical performance measured by a gait speed of ≤0.8 m/s. Although the two components were associated with the risk of disability, the definition using ≤0.8 m/s as the cut-off point for low gait speed exhibited a greater effect size for the risk of disability in both BADL (16%) and IADL (19%).

No prior study has compared slowness, as defined by the frailty phenotype, with the low physical performance associated with the sarcopenia construct. Although the literature consistently supports the predictive power of low gait speed regarding adverse outcomes in older adults, this stands true irrespective of the adopted cut-off point [12, 43, 50–52]. The mechanisms underlying low physical performance include joint diseases, exhaustion or fatigue, reduced muscle strength, sarcopenia and low levels of physical activity [49–51], many of which are components of both frailty and sarcopenia. Furthermore, in addition to assessing mobility, often regarded as the sixth vital sign in older adults [53, 54], we propose that it may be possible to identify the risk of disability by analysing gait speed alone. Therefore, considering the similarity in the decline of the domains suggested by Rivera, which are present in both constructs, low physical performance appears to be a component of frailty that can capture a significant part of the homeostatic imbalance within the syndrome.

Thus, gait speed- a low-cost measure that is easy to apply in clinical practice- may be the best means of identifying the risk of disability in older adults’ activities of daily living.

The present study has both limitations and strengths that should be acknowledged. Our findings should be viewed in the context of community-dwelling older adults (≥60 years of age) who do not have disabilities regarding BADL and IADL. Therefore, caution must be exercised when extrapolating the results to clinical or hospital settings, as well as assisted living facilities. Another significant limitation relates to determining skeletal muscle mass through an equation. However, this does not diminish our findings, as the equation has strong construct validity coefficients based on validation with one of the ‘gold standard’ methods, such as magnetic resonance, and provides a more practical means of estimating muscle mass in clinical contexts with limited resources. The differences in baseline characteristics between participants who remained throughout the follow-up period and those lost to follow-up during the first 4 or 8 years may increase the risk of selection bias in our results. We employed inverse probability weighting, a robust statistical strategy that significantly reduces such bias [38–41]. This study also has strengths, such as using standard tools to identify the frailty phenotype, encompassing a large, representative sample of community-dwelling individuals in England and an 8-year follow-up period. Furthermore, this is the first study to compare sarcopenia as defined by the *EWGSOP2* criteria with frailty and the individual components of each condition, in models adjusted for a wide range of covariates associated with disability.

## Conclusion

Both the frailty phenotype and low physical performance, defined by a gait speed of ≤0.8 m/s, are strong predictors of the incidence of functional disability in individuals aged 60 and older. However, considering ease of use, agility, low cost and clinical applicability across various healthcare scenarios, gait speed with a cut-off of ≤0.8 m/s for identifying low physical performance should be prioritised in clinical practice for developing prevention strategies aimed at avoiding the onset of disability related to BADL and IADL in older adults.

From a practical standpoint, conducting two consecutive trials over a distance of 2.4 m on a flat surface is advisable for standardising the measurement of gait speed in older adults. The total distance in metres is divided by the time in seconds to convert the final result into metres per second (m/s). The literature indicates that this short distance (2.4 m) is as effective as longer distances in predicting adverse outcomes and allows the test to be conducted in smaller spaces [55–57]. This standardisation enables clinicians to incorporate the assessment of gait speed in various care settings for older adults [58].

## Supplementary Material

aa-24-2168-File002afaf_104
