# Supplementary material for: Low gait speed is better than frailty and sarcopenia at identifying the risk of disability in older adults
Source: Age Ageing. 2025 Apr 23;54(4):afaf104. doi: 10.1093/ageing/afaf104 (PMC12017394; doi:10.1093/ageing/afaf104)
Supplement: aa-24-2168-File002afaf_104 [file aa-24-2168-file002afaf_104.docx]

**Low physical performance is better than frailty and sarcopenia for identifying the risk of disability in older adults**

**Supplementary material**

1. **Supplementary methods**

Frailty assessment

Sarcopenia Assessment

Basic Activities of Daily Living Assessment

Instrumental Activities of Daily Living Assessment

Covariates Assessment

1. **Supplementary tables**

Supplementary table 1. Cut-off points used for defining frailty components muscle weakness and slowness stratified by sex, ELSA study 2004/2005.

Supplementary Table 2. Comparison of socioeconomic characteristics, behavioural characteristics, health conditions, anthropometrics, frailty, sarcopenia and respective components of frailty at baseline between participants who remained throughout the entire follow-up period and those lost to follow-up in first four years or entire eight-year period with regards to incidence of disability related to BADL, ELSA Study (2004).

Supplementary Table 3. Comparison of socioeconomic characteristics, behavioural characteristics, health conditions, anthropometrics, frailty, sarcopenia and respective components of frailty at baseline between participants who remained throughout entire follow-up period and those lost to follow-up in first four years or entire eight-year period with regards to incidence of disability related to IADL, ELSA Study (2004).

1. **Supplementary figures**

Supplementary Figure 1. Incidence of disability on BADL and IADL as a function of frailty phenotype, sarcopenia and physical performance over eight-year follow-up – English Longitudinal Study of Ageing 2004/2005 – 2012/2013. Models adjusted by age, sex, race, marital status, schooling, total family wealth, smoking, alcohol intake, physical activity, systemic arterial hypertension, diabetes mellitus, cancer, lung disease, heart disease, stroke, osteoarthritis, osteoporosis, falls, depressive symptoms, memory score and BMI.

Supplementary Figure 2. Incidence of disability on BADL according to individual components of sarcopenia construct over eight-year follow-up – English Longitudinal Study of Ageing 2004/2005 – 2012/2013. Model adjusted by age, sex, race, marital status, schooling, total family wealth, smoking, alcohol intake, physical activity, systemic arterial hypertension, diabetes mellitus, cancer, lung disease, heart disease, stroke, osteoarthritis, osteoporosis, falls, depressive symptoms, memory score and BMI.

Supplementary Figure 3. Incidence of disability on IADL according to individual components of sarcopenia construct over eight-year follow-up – English Longitudinal Study of Ageing 2004/2005 – 2012/2013. Model adjusted by age, sex, race, marital status, schooling, total family wealth, smoking, alcohol intake, physical activity, systemic arterial hypertension, diabetes mellitus, cancer, lung disease, heart disease, stroke, osteoarthritis, osteoporosis, falls, depressive symptoms, memory score and BMI.

1. **Supplementary references**
2. **Supplementary methods**

**Frailty assessment**

Frailty was measured by the phenotype (2001) [1]. Unintentional weight loss was defined by a body mass index (BMI) of < 18. 5 kg/m ² [1, 2]. Exhaustion was defined by an affirmative answer to either of the following two statements from the *Center for Epidemiologic Studies Depression Scale* (*CES- D*): "I felt that everything I did was an effort in the past week" or "I could not get 'going' in the past week" [1, 3]. Muscle strength was estimated based on grip strength, while muscle weakness was defined by the lowest quintile of grip strength stratified by sex in each BMI quartile [1]. This resulted in cut- off points of ≤ 31, ≤ 33, ≤ 34, and ≤ 35 kg for men, and ≤ 18, ≤ 19, and ≤ 20 kg for women (Supplementary table 1). Participants were instructed to walk 2. 4 meters on a flat surface without assistance to determine gait speed, with the better time of two trials recorded. Slowness was defined as the lowest gait speed quintile based on the average of two measures (2. 4 m) stratified by average height for each sex [1] (1. 72 m for men and 1. 59 m for women), resulting in a cut-off point of < 3. 56 seconds for men and < 4. 25 seconds for women. Low physical activity level was determined using three questions from the Physical Activity and Sedentary Behaviour Questionnaire (PASBAQ) used in the *Health Survey for England* (*HSE*), taking into account the frequency and intensity with which participants practised vigorous, moderate, and light physical activities (more than once per month, once per week, one to three times per week, or never). Those who reported never participating in moderate- intensity physical activity were considered to have a low physical activity level [4-6]. Participants with three or more frailty components were considered frail, those with one or two components were deemed pre- frail, and those with no components were classified as non- frail. In addition to the complete frailty construct, each element was analysed individually as a dichotomous variable (present or absent).

**Sarcopenia Assessment**

Sarcopenia was defined according to the *EWGSOP2* [7]*.* Muscle strength was estimated using grip strength measured with a handgrip dynamometer (Smedley; range: 0 to 100 kg). The test was conducted while the participant stood, with arms alongside the body and elbows flexed at 90 degrees. Three trials were done with the dominant hand, allowing for a one-minute rest between each trial. The highest value was used for the analyses [6,8,9]. The grip strength cut-off points defining low muscle strength were < 27 kg for men and < 16 kg for women [7].

Skeletal Muscle Mass (SMM) was determined using the equation proposed by Lee [10]. Al-Gindan and colleagues (2014) validated this equation using whole-body magnetic resonance imaging and found an adjusted coefficient of determination of 0.85 for both men and women [11]. Spexoto and colleagues (2022) also employed this equation to estimate SMM in a study examining the association between mortality and various cut-off points for sarcopenia over a 14-year period [12]. Following the estimation of SMM, the Skeletal Muscle Mass Index (SMMI) (kg/m^²^) was calculated. The cut-off points for defining low SMMI were based on the 20^th^ percentile of the sample distribution [13-14]. The SMMI values used to define low muscle mass were < 9.36 kg/m^²^ for men and < 6.73 kg/m^²^ for women.

Physical performance was evaluated based on gait speed. The total distance in meters was divided by the time in seconds (m/s). Low physical performance was defined as a gait speed of ≤ 0.8 m/s [7,15,16].

For the sarcopenia construct, the participants were classified into four groups: no sarcopenia, probable sarcopenia (only low strength), sarcopenia (low strength + low muscle mass), or severe sarcopenia (low strength + low mass + low physical performance).^7^ In addition to the complete sarcopenia construct, each component was also considered individually for the analyses as a dichotomous variable (present or absent).

**Basic Activities of Daily Living Assessment**

The basic activities of daily living were assessed using the modified Katz index [17] (walking, bathing, toileting, dressing, feeding oneself, and lying down and getting up from bed). Although continence is one of the items in the index, it does not necessarily indicate a physical limitation and was, therefore, excluded from the current analysis [18]. Only individuals who experienced no difficulties regarding any BADL at baseline were included. The incidence of difficulties with BADL during the eight-year follow-up period was analysed, with scores ranging from 0 to 6 in the subsequent waves of the *ELSA* study.

### Instrumental Activities of Daily Living Assessment

The instrumental activities of daily living were assessed using the adapted Lawton scale [19], which includes preparing meals, shopping, managing finances, using the telephone, managing medications, and performing household chores. Only individuals who experienced no difficulties with any IADL at baseline were included. The incidence of difficulties with IADL during the eight-year follow-up period was analysed, with scores ranging from 0 to 6 in the subsequent waves of the *ELSA* study.

**Covariates Assessment**

The variables described in the literature as associated with disability were considered control variables and measured at the present study's baseline (2004). The sociodemographic variables were age (continuous), sex, race (white or non-white), marital status (with or without a conjugal life), schooling following the English standard (0–11; 12–13; > 13 years), and total family wealth (quintiles) [20,21].

The behavioural characteristics of interest included smoking (non-smoker, former smoker, or smoker) and alcohol intake (non-drinker, intake up to once per week, intake two to six times per week, or daily intake) [20]. Physical activity levels were assessed based on responses to three questions from the Physical Activity and Sedentary Behaviour Questionnaire (PASBAQ) used in the *Health Survey for England* (*HSE*), considering the frequency and intensity of the participant’s physical activity. Participants reported the frequency (once per week, more than once per week, one to three times per month, or hardly ever/never) of vigorous exercises (e.g., running, swimming, cycling, tennis, and weightlifting), moderate exercises (e.g., gardening, washing the car, walking at a moderate pace, dancing, and stretching), and light exercises (e.g., vacuuming, washing clothes, and doing household repairs). Physical activity was categorised as vigorous (engaging in vigorous activity at least once a week), moderate (engaging in moderate activity at least once a week), or light (only light activity at least once a week). Individuals with no weekly activity were inactive [4-6].

Health conditions were assessed based on self-reports of systemic arterial hypertension, diabetes mellitus, cancer, lung disease, heart disease, stroke, osteoarthritis, osteoporosis, and the occurrence of falls in the previous year. Depressive symptoms were defined by a score of ≥ 4 points on the *Center for Epidemiological Studies-Depression Scale* [22]. Memory was evaluated using the word list test, which is divided into two parts: immediate recall (the participant hears 10 words and immediately repeats them) and delayed recall (the participant is asked to recall the words after three minutes). The score is determined by the number of correctly recalled words in both parts of the test and ranges from 0 to 20, with higher scores indicating better memory performance [23]. For body mass index, individuals were classified as eutrophic (≥ 18.5 BMI < 25 kg/m²), underweight (BMI < 18.5 kg/m²), overweight (≥ 25 BMI < 30 kg/m²), or obese (BMI ≥ 30 kg/m²) [2].

1. **Supplementary tables**

**Supplementary table 1.** Cut-off points used for defining frailty components muscle weakness and slowness stratified by sex, ELSA study 2004/2005.

| **Component** | **Definition** |
| --- | --- |
| **Muscle weakness** | 20^th^ percentile of sample distribution for grip strength by sex and BMI quartile |
|  | **Men** |
|  | Grip strength ≤ 31 kg for BMI ≤ 25.10 kg/m² |
|  | Grip strength ≤ 33 kg for BMI 25.10 ≤ 27.32 kg/m² |
|  | Grip strength ≤ 34 kg for BMI 27.32 ≤ 30.19 kg/m² |
|  | Grip strength ≤ 35 kg for BMI > 30.19 kg/m² |
|  |  |
|  | **Women** |
|  | Grip strength ≤ 18 kg for BMI ≤ 24.16 kg/m² |
|  | Grip strength ≤ 19 kg for BMI 24.16 ─ 27.14 kg/m² |
|  | Grip strength ≤ 20 kg for BMI 27.14 ─ 31.03 kg/m² |
|  | Grip strength ≤ 19 kg for BMI > 31.03 kg/m² |
|  |  |
| **Slowness** | 20^th^ percentile of sample distribution for gait speed by sex and average height |
|  | **Men** |
|  | > 3.56 seconds for height ≤ 1.72 m |
|  | > 3.24 seconds for height > 1.72 m |
|  |  |
|  | **Women** |
|  | > 4.22 seconds for height ≤ 1.59 m |
|  | > 3.39 seconds for height > 1.59 m |

Note: BMI: body mass index.

**Supplementary Table 2.** Comparison of socioeconomic characteristics, behavioural characteristics, health conditions, anthropometrics, frailty, sarcopenia and respective components of frailty at baseline between participants who remained throughout the entire follow-up period and those lost to follow-up in first four years or entire eight-year period with regards to incidence of disability related to BADL, ELSA Study (2004).

|  | **BADL** | | | |
| --- | --- | --- | --- | --- |
|  | **Total n = 3,637** | | **Total n = 3,637** | |
|  | **Followed for 4 years n = 2,696** | **Lost in 4 years**  **n = 941** | **Followed for 8 years n = 2,026** | **Lost in 8 years**  **n = 1,611** |
| **Socioeconomic characteristics** |  |  |  |  |
| Age, (mean ± SD) | (n = 2,696) 69.4 ± 6.9 | (n = 941) 71.5 ± 7.8^a^ | (n = 2,026) 68.3 ± 6.3 | (n = 1,611) 71.9 ± 7.8^b^ |
| Sex (female), % | (n = 1,476) 54.8 | (n = 494) 52.5 | (n = 1,142) 56.4 | (n = 828) 51.4^b^ |
| Race (white), % | (n = 2,657) 99.0 | (n = 924) 98.2 | (n = 1,998) 98.6 | (n = 1,583) 98.3 |
| Marital status (without conjugal life), % | (n = 891) 33.1 | (n = 294) 31.2 | (n = 613) 30.3 | (n = 572) 35.5^b^ |
| Schooling, % |  |  |  |  |
| >13 years | (n = 695) 25.8 | (n = 161) 17.1^a^ | (n = 568) 28.0 | (n = 288) 17.9^b^ |
| 12 – 13 years | (n = 626) 23.2 | (n = 167) 17.8^a^ | (n = 494) 24.4 | (n = 299) 18.5^b^ |
| 0 – 11 years | (n = 1,375) 51.0 | (n = 613) 65.1^a^ | (n = 964) 47.6 | (n = 1,024) 63.6^b^ |
| Total family wealth (quintiles), % |  |  |  |  |
| Fifth quintile (highest) | (n = 693) 25.7 | (n = 188) 20.0^a^ | (n = 578) 28.5 | (n = 303) 18.8^b^ |
| Fourth quintile | (n = 609) 22.6 | (n = 194) 20.6 | (n = 464) 22.9 | (n = 339) 21.1 |
| Third quintile | (n = 550) 20.4 | (n = 189) 20.1 | (n = 417) 20.6 | (n = 322) 20.0 |
| Second quintile | (n = 470) 17.4 | (n = 194) 20.6 | (n = 326) 16.1 | (n = 338) 21.0^b^ |
| First quintile (lowest) | (n = 341) 12.7 | (n = 170) 18.1^a^ | (n = 222) 11.0 | (n = 289) 17.9^b^ |
| Not declared | (n = 33) 1.2 | (n = 6) 0.6 | (n = 19) 0.9 | (n = 20) 1.2 |
| **Behavioural characteristics** |  |  |  |  |
| Smoking, % |  |  |  |  |
| Non-smoker | (n = 1,069) 39.7 | (n = 306) 32.5^a^ | (n = 829) 40.9 | (n = 546) 33.9^b^ |
| Former smoker | (n = 1,340) 49.7 | (n = 499) 53.0 | (n = 1,010) 49.9 | (n = 829) 51.5 |
| Smoker | (n = 287) 10.6 | (n = 136) 14.5^a^ | (n = 187) 9.2 | (n = 236) 14.6^b^ |
| Alcohol intake, % |  |  |  |  |
| Non-drinker or intake up to once per week | (n = 449) 16.7 | (n = 176) 18.7 | (n = 325) 16.0 | (n = 300) 18.6 |
| Intake two to six times per week | (n = 1,133) 42.0 | (n = 361) 38.4 | (n = 880) 43.4 | (n = 614) 38.1^b^ |
| Daily intake | (n = 924) 34.3 | (n = 280) 29.7^a^ | (n = 704) 34.8 | (n = 500) 31.1 |
| Not declared | (n = 190) 7.0 | (n = 124) 13.2^a^ | (n = 117) 5.8 | (n = 197) 12.2^b^ |
| Physical activity, % |  |  |  |  |
| Vigorous | (n = 5) 0.2 | (n = 0) 0.0 | (n = 4) 0.2 | (n = 1) 0.1 |
| Moderate | (n = 43) 1.6 | (n = 25) 2.7 | (n = 38) 1.9 | (n = 30 )1.9 |
| Light | (n = 2,594) 96.2 | (n = 883) 93.8 | (n = 1,956) 96.5 | (n = 1,521) 94.4^b^ |
| Inactive | (n = 54) 2.0 | (n = 33) 3.5 | (n = 28) 1.4 | (n = 59) 3.6^b^ |
| *continue* |  |  |  |  |
| **Health conditions** |  |  |  |  |
| Systemic arterial hypertension, (yes) % | (n = 1,175) 43.6 | (n = 459) 48.8^a^ | (n = 848) 41.9 | (n = 786) 48.8^b^ |
| Diabetes mellitus (yes), % | (n = 195) 7.2 | (n = 81) 8.6 | (n = 139) 6.9 | (n = 137) 8.5 |
| Cancer (yes), % | (n = 223) 8.3 | (n = 108) 11.5^a^ | (n = 162) 8.0 | (n = 169) 10.5^b^ |
| Lung disease (yes), % | (n = 422) 15.7 | (n = 182) 19.3^a^ | (n = 319) 15.8 | (n = 285) 17.7^b^ |
| Heart disease (yes), % | (n = 587) 21.8 | (n = 235) 25.0^a^ | (n = 404) 19.9 | (n = 418) 26.0^b^ |
| Stroke (yes), % | (n = 92) 3.4 | (n = 54) 5.7^a^ | (n = 63) 3.1 | (n = 83) 5.2^b^ |
| Osteoarthritis (yes), % | (n = 914) 33.9 | (n = 319) 33.9 | (n = 676) 33.4 | (n = 557) 34.6 |
| Osteoporosis (yes), % | (n = 168) 6.2 | (n = 60) 6.4 | (n = 123) 6.1 | (n = 105) 6.5 |
| Falls (yes), % | (n = 740) 27.5 | (n = 247) 26.3 | (n = 542) 26.7 | (n = 445) 27.6 |
| Depressive symptoms (yes), % | (n = 259) 9.6 | (n = 112) 11.9 | (n = 186) 9.2 | (n = 185) 11.5 |
| Memory score, (mean - SD) | (n = 2,696) 10.0 ± 3.2 | (n = 941) 8.7 ± 3.6^a^ | (n = 2,026) 10.3 ± 3.0 | (n = 1,611) 8.9 ± 3.5^b^ |
| **Anthropometry** |  |  |  |  |
| Body mass index, % |  |  |  |  |
| Eutrophic (≥ 18.5 kg/m² BMI < 25 kg/m²) | (n = 781) 29.0 | (n = 269) 28.6 | (n = 579) 28.6 | (n = 471) 29.2 |
| Underweight (<18.5 kg/m²) | (n = 15) 0.5 | (n = 17) 1.8^a^ | (n = 8) 0.4 | (n = 24) 1.5^b^ |
| Overweight (≥ 25 kg/m² BMI < 30 kg/m²) | (n = 1,267) 47.0 | (n = 412) 43.8 | (n = 954) 47.1 | (n = 725) 45.0 |
| Obesity (≥ 30 kg/m²) | (n = 633) 23.5 | (n = 243) 25.8 | (n = 485) 23.9 | (n = 391) 24.3 |
| **Frailty** |  |  |  |  |
| Non-frail, % | (n = 1,545) 57.3 | (n = 411) 43.7^a^ | (n = 1,232) 60.8 | (n = 724) 44.9^b^ |
| Pre-frail, % | (n = 1,066) 39.5 | (n = 424) 45.1^a^ | (n = 748) 36.9 | (n = 742) 46.1^b^ |
| Frail, % | (n = 85) 3.2 | (n = 106) 11.2^a^ | (n = 46) 2.3 | (n = 145) 9.0^b^ |
| **Components of frailty** |  |  |  |  |
| Unintentional weight loss (yes), % | (n = 15) 0.6 | (n = 17) 1.8^a^ | (n = 8) 0.4 | (n = 24) 1.5^b^ |
| Exhaustion (yes), % | (n = 525) 19.5 | (n = 252) 26.8^a^ | (n = 372) 18.4 | (n = 405) 25.1^b^ |
| Weakness (yes), % | (n = 550) 20.4 | (n = 305) 32.4^a^ | (n = 365) 18.0 | (n = 490) 30.4^b^ |
| Slowness (yes), % | (n = 281) 10.4 | (n = 185) 19.7^a^ | (n = 185) 9.1 | (n = 281) 17.4^b^ |
| Low physical activity level (yes), % | (n = 225) 8.4 | (n = 167) 17.8^a^ | (n = 134) 6.6 | (n = 258) 16.0^b^ |
| **Sarcopenia** |  |  |  |  |
| No sarcopenia, % | (n = 2,540) 94.2 | (n = 838) 89.1^a^ | (n = 1,925) 95.0 | (n = 1,453) 90.2^b^ |
| Probable sarcopenia, % | (n = 93) 3.5 | (n = 47) 5.0 | (n = 63) 3.1 | (n = 77) 4.8 |
| Sarcopenia, % | (n = 25) 0.9 | (n = 20) 2.1^a^ | (n = 18) 0.9 | (n = 27) 1.7 |
| Severe sarcopenia, % | (n = 38) 1.4 | (n = 36) 3.8^a^ | (n = 20) 1.0 | (n = 54) 3.3^b^ |
| **Components of sarcopenia** |  |  |  |  |
| Muscle strength (< 27/16 kg), % | (n = 156) 5.8 | (n = 103) 11.0^a^ | (n = 101) 5.0 | (n = 158) 9.8^b^ |
| Skeletal muscle mass index (< 9.36/6.73 kg/m^2^), % | (n = 639) 23.7 | (n = 263) 28.0^a^ | (n = 423) 20.9 | (n = 479) 29.7^b^ |
| Physical performance (≤ 0.8 m/s), % | (n = 678) 25.2 | (n = 352) 37.4^a^ | (n = 435) 21.5 | (n = 595) 36.9^b^ |

Note: Data is expressed in proportions, as well as means and standard deviations. ^a^comparison of baseline characteristics between participants followed up in first four years and those lost to follow-up in this period (n = 2,696 versus 941); ^b^ comparison of baseline characteristics between participants followed up in entire eight-year period and those lost to follow-up in this period (n = 2,026 versus 1,611).

**Supplementary Table 3.** Comparison of socioeconomic characteristics, behavioural characteristics, health conditions, anthropometrics, frailty, sarcopenia and respective components of frailty at baseline between participants who remained throughout entire follow-up period and those lost to follow-up in first four years or entire eight-year period with regards to incidence of disability related to IADL, ELSA Study (2004).

|  | **IADL** | | | |
| --- | --- | --- | --- | --- |
|  | **Total n = 3,696** | | **Total n = 3,696** | |
|  | **Followed for 4 years n = 2,779** | **Lost in 4 years**  **n = 917** | **Followed for 8 years n = 2,100** | **Lost in 8 years**  **n = 1,596** |
| **Socioeconomic characteristics** |  |  |  |  |
| Age, (mean - SD) | (n = 2,779) 69.4 ± 6.8 | (n = 917) 70.9 ± 7.5^a^ | (n = 2,100) 68.3 ± 6.2 | (n = 1,596) 71.6 ± 7.5^b^ |
| Sex (female), % | (n = 1,496) 53.8 | (n = 464) 50.6 | (n = 1,155) 55.0 | (n = 805) 50.4^b^ |
| Race (white), % | (n = 2,741) 98.6 | (n = 901) 98.3 | (n = 2,072) 98.7 | (n = 1,570) 98.4 |
| Marital status (without conjugal life), % | (n = 904) 32.5 | (n = 267) 29.1 | (n = 618) 29.4 | (n = 553) 34.7^b^ |
| Schooling, % |  |  |  |  |
| >13 years | (n = 717) 25.8 | (n = 159) 17.3^a^ | (n = 587) 28.0 | (n = 289) 18.1^b^ |
| 12 – 13 years | (n = 656) 23.6 | (n = 171) 18.7^a^ | (n = 517) 24.6 | (n = 310) 19.4^b^ |
| 0 – 11 years | (n = 1,406) 50.6 | (n = 587) 64.0^a^ | (n = 996) 47.4 | (n = 997) 62.5^b^ |
| Total family wealth (quintiles), % |  |  |  |  |
| Fifth quintile (highest) | (n = 704) 25.3 | (n = 180) 19.6^a^ | (n = 584) 27.8 | (n = 300) 18.8^b^ |
| Fourth quintile | (n = 636) 22.9 | (n = 189) 20.6 | (n = 489) 23.3 | (n = 336) 21.1 |
| Third quintile | (n = 583) 21.0 | (n = 197) 21.5 | (n = 445) 21.2 | (n = 335) 21.0 |
| Second quintile | (n = 484) 17.4 | (n = 174) 19.0 | (n = 336) 16.0 | (n = 322) 20.2^b^ |
| First quintile (lowest) | (n = 336) 12.1 | (n = 170) 18.5^a^ | (n = 225) 10.7 | (n = 281) 17.6^b^ |
| Not declared | (n = 36) 1.3 | (n = 7) 0.8 | (n = 21) 1.0 | (n = 22) 1.3 |
| **Behavioural characteristics** |  |  |  |  |
| Smoking, % |  |  |  |  |
| Non-smoker | (n = 1,089) 39.2 | (n = 300) 32.7^a^ | (n = 848) 40.4 | (n = 541) 33.9^b^ |
| Former smoker | (n = 1,409) 50.7 | (n = 488) 53.2 | (n = 1,055) 50.2 | (n = 842) 52.8 |
| Smoker | (n = 281) 10.1 | (n = 129) 14.1^a^ | (n = 197) 9.4 | (n = 213) 13.3^b^ |
| Alcohol intake, % |  |  |  |  |
| Non-drinker or intake up to once per week | (n = 447) 16.1 | (n = 171) 18.7 | (n = 331) 15.8 | (n = 287) 18.0 |
| Intake two to six times per week | (n = 1,183) 42.6 | (n = 359) 39.1 | (n = 927) 44.1 | (n = 615) 38.5^b^ |
| Daily intake | (n = 956) 34.4 | (n = 277) 30.2^a^ | (n = 727) 34.6 | (n = 506) 31.7 |
| Not declared | (n = 193) 6.9 | (n = 110) 12.0^a^ | (n = 115) 5.5 | (n = 188) 11.8^b^ |
| Physical activity, % |  |  |  |  |
| Vigorous | (n = 5) 0.2 | (n = 0) 0.0 | (n = 4) 0.2 | (n = 1) 0.1 |
| Moderate | (n = 46) 1.7 | (n = 23) 2.5 | (n = 40) 1.9 | (n = 29) 1.8 |
| Light | (n = 2,683) 96.5 | (n = 869) 94.8 | (n = 2,032) 96.8 | (n = 1,520) 95.2^b^ |
| Inactive | (n = 45) 1.6 | (n = 25) 2.7 | (n = 24) 1.1 | (n = 46) 2.9^b^ |
| *continue* |  |  |  |  |
| **Health conditions** |  |  |  |  |
| Systemic arterial hypertension, (yes) % | (n = 1,220) 43.9 | (n = 459) 50.1^a^ | (n = 885) 42.1 | (n = 794) 49.8^b^ |
| Diabetes mellitus (yes), % | (n = 206) 7.4 | (n = 83) 9.1 | (n = 142) 6.8 | (n = 147) 9.2 |
| Cancer (yes), % | (n = 231) 8.3 | (n = 94) 10.3^a^ | (n = 164) 7.8 | (n = 161) 10.1^b^ |
| Lung disease (yes), % | (n = 424) 15.3 | (n = 165) 18.0^a^ | (n = 313) 14.9 | (n = 276) 17.3^b^ |
| Heart disease (yes), % | (n = 593) 21.3 | (n = 218) 23.8^a^ | (n = 415) 19.8 | (n = 396) 24.8^b^ |
| Stroke (yes), % | (n = 83) 3.0 | (n = 45) 4.9^a^ | (n = 64) 3.1 | (n = 64) 4.0^b^ |
| Osteoarthritis (yes), % | (n = 973) 35.0 | (n = 310) 33.8 | (n = 726) 34.6 | (n = 557) 34.9 |
| Osteoporosis (yes), % | (n = 171) 6.2 | (n = 58) 6.3 | (n = 126) 6.0 | (n = 103) 6.5 |
| Falls (yes), % | (n = 763) 27.5 | (n = 233) 25.4 | (n = 560) 26.7 | (n = 436) 27.3 |
| Depressive symptoms (yes), % | (n = 263) 9.5 | (n = 96) 10.5 | (n = 195) 9.3 | (n = 164) 10.3 |
| Memory score, (mean - SD) | (n = 2,779) 10.0 ± 3.1 | (n = 917) 8.7 ± 3.6^a^ | (n = 2,100) 10.3 ± 3.0 | (n = 1,596) 8.9 ± 3.5^b^ |
| **Anthropometry** |  |  |  |  |
| Body mass index, % |  |  |  |  |
| Eutrophic (≥ 18.5 kg/m² BMI < 25 kg/m²) | (n = 774) 27.9 | (n = 248) 27.1 | (n = 579) 27.6 | (n = 443) 27.8 |
| Underweight (<18.5 kg/m²) | (n = 12) 0.4 | (n = 16) 1.7^a^ | (n = 7) 0.3 | (n = 21) 1.3^b^ |
| Overweight (≥ 25 kg/m² BMI < 30 kg/m²) | (n = 1,295) 46.6 | (n = 391) 42.6 | (n = 977) 46.5 | (n = 709) 44.4 |
| Obesity (≥ 30 kg/m²) | (n = 698) 25.1 | (n = 262) 28.6 | (n = 537) 25.6 | (n = 423) 26.5 |
| **Frailty** |  |  |  |  |
| Non-frail, % | (n = 1,598) 57.5 | (n = 423) 46.1^a^ | (n = 1,279) 60.9 | (n = 742) 46.5^b^ |
| Pre-frail, % | (n = 1,099) 39.5 | (n = 407) 44.4^a^ | (n = 772) 36.8 | (n = 734) 46.0^b^ |
| Frail, % | (n = 82) 3.0 | (n = 87) 9.5^a^ | (n = 49) 2.3 | (n = 120) 7.5^b^ |
| **Components of frailty** |  |  |  |  |
| Unintentional weight loss (yes), % | (n = 12) 0.4 | (n = 16) 1.7^a^ | (n = 7) 0.3 | (n = 21) 1.3^b^ |
| Exhaustion (yes), % | (n = 544) 19.6 | (n = 227) 24.8^a^ | (n = 390) 18.6 | (n = 381) 23.9^b^ |
| Weakness (yes), % | (n = 569) 20.5 | (n = 277) 30.2^a^ | (n = 380) 18.1 | (n = 466) 29.2^b^ |
| Slowness (yes), % | (n = 289) 10.4 | (n = 168) 18.3^a^ | (n = 193) 9.2 | (n = 264) 16.5^b^ |
| Low physical activity level (yes), % | (n = 203) 7.3 | (n = 131) 14.3^a^ | (n = 120) 5.7 | (n = 214) 13.4^b^ |
| **Sarcopenia** |  |  |  |  |
| No sarcopenia, % | (n = 2,618) 94.2 | (n = 823) 89.8^a^ | (n = 1,997) 95.1 | (n = 1,444) 90.5^b^ |
| Probable sarcopenia, % | (n = 104) 3.7 | (n = 43) 4.7 | (n = 70) 3.3 | (n = 77) 4.8 |
| Sarcopenia, % | (n = 24) 0.9 | (n = 17) 1.8^a^ | (n = 15) 0.7 | (n = 26) 1.6 |
| Severe sarcopenia, % | (n = 33) 1.2 | (n = 34) 3.7^a^ | (n = 18) 0.9 | (n = 49) 3.1^b^ |
| **Components of sarcopenia** |  |  |  |  |
| Muscle strength (< 27/16 kg), % | (n = 161) 5.8 | (n = 94) 10.3^a^ | (n = 103) 4.9 | (n = 152) 9.5^b^ |
| Skeletal muscle mass index (< 9.36/6.73 kg/m^2^), % | (n = 629) 22.6 | (n = 230) 25.1^a^ | (n = 426) 20.3 | (n = 433) 29.1^b^ |
| Physical performance (≤ 0.8 m/s), % | (n = 688) 24.8 | (n = 326) 35.6^a^ | (n = 447) 21.3 | (n = 567) 35.5^b^ |

Note: Data is expressed in proportions, as well as means and standard deviations. ^a^ comparison of baseline characteristics between participants followed up in the first four years and those lost to follow-up in this period (n = 2,779 versus 917); ^b^ comparison of baseline characteristics between participants followed up in the entire eight-year period and those lost to follow-up in this period (n = 2,100 versus 1,596).

1. **Supplementary figures**


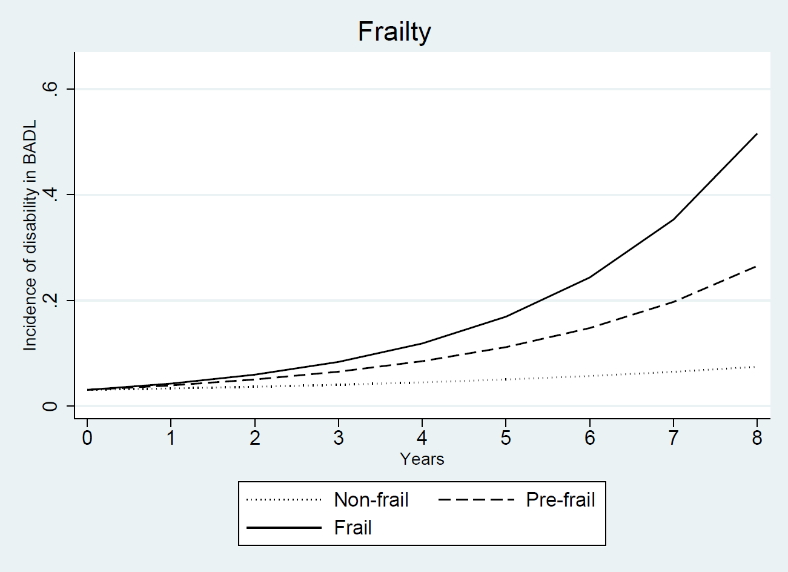

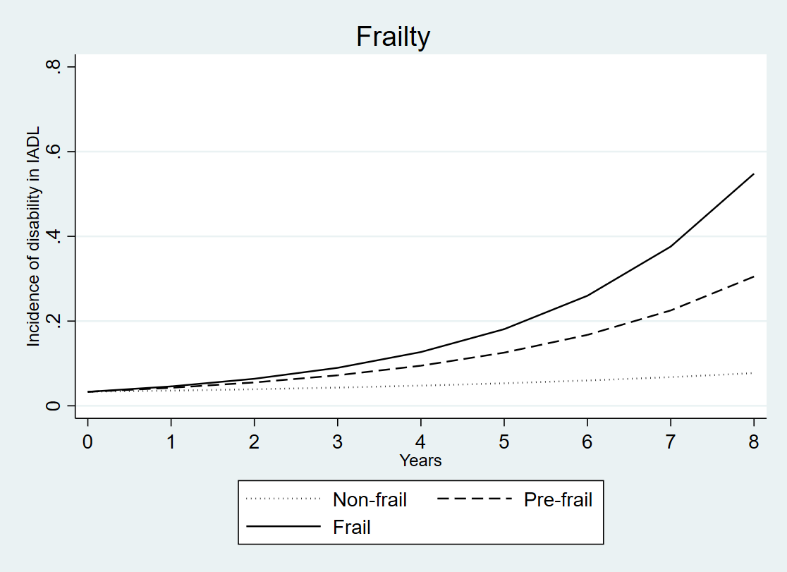

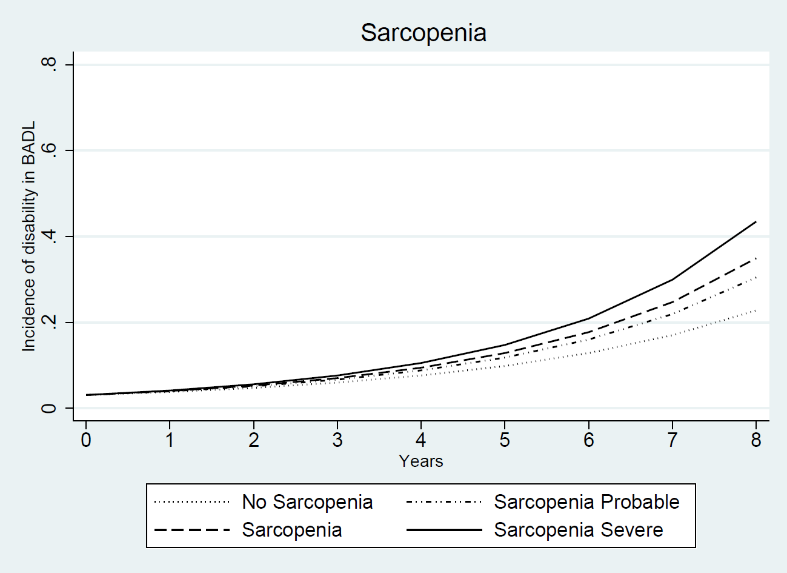

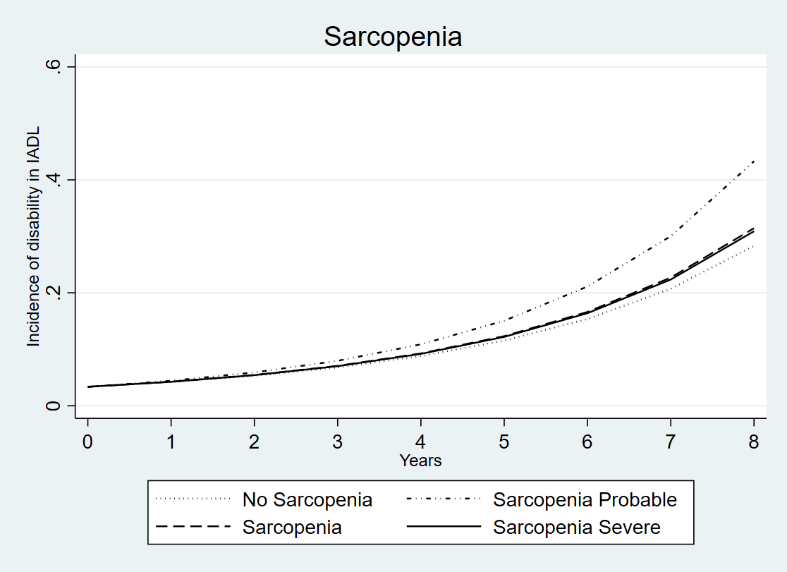


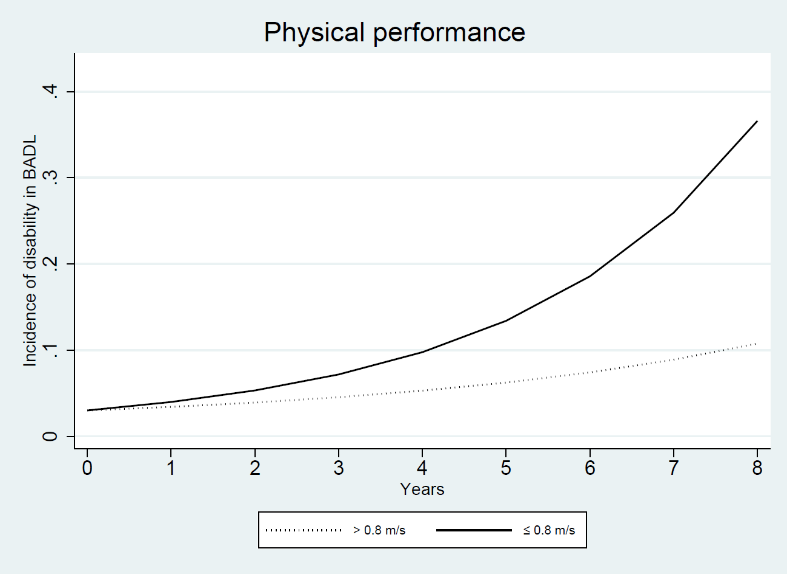

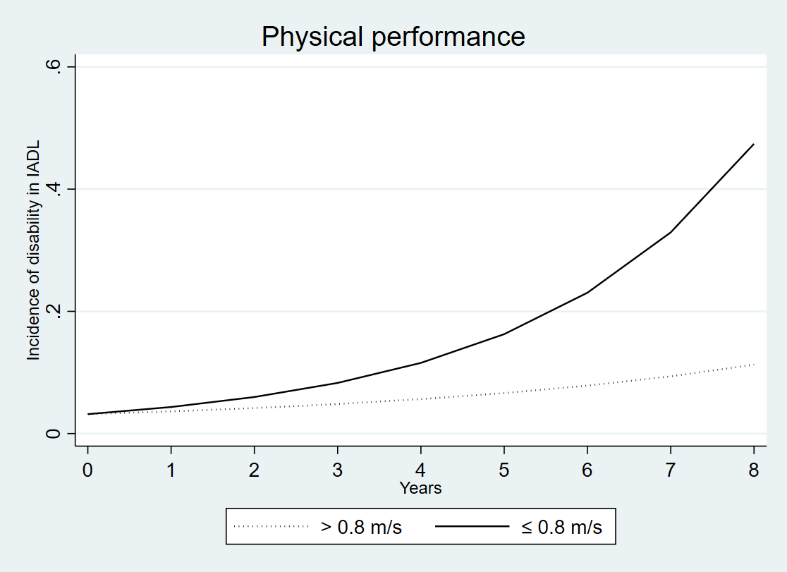


**Supplementary Figure 1.** Incidence of disability on BADL and IADL as a function of frailty phenotype, sarcopenia and physical performance over eight-year follow-up – *English Longitudinal Study of Ageing* 2004/2005 – 2012/2013. Models adjusted by age, sex, race, marital status, schooling, total family wealth, smoking, alcohol intake, physical activity, systemic arterial hypertension, diabetes mellitus, cancer, lung disease, heart disease, stroke, osteoarthritis, osteoporosis, falls, depressive symptoms, memory score and BMI.


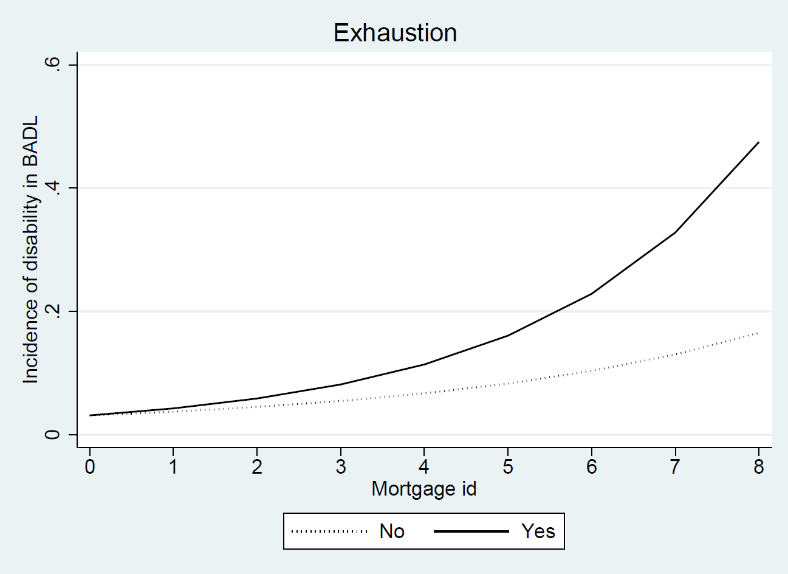

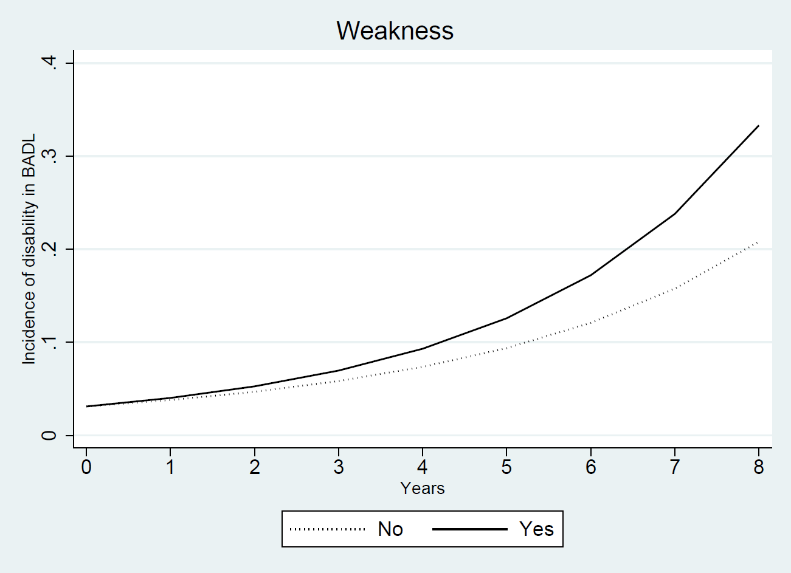


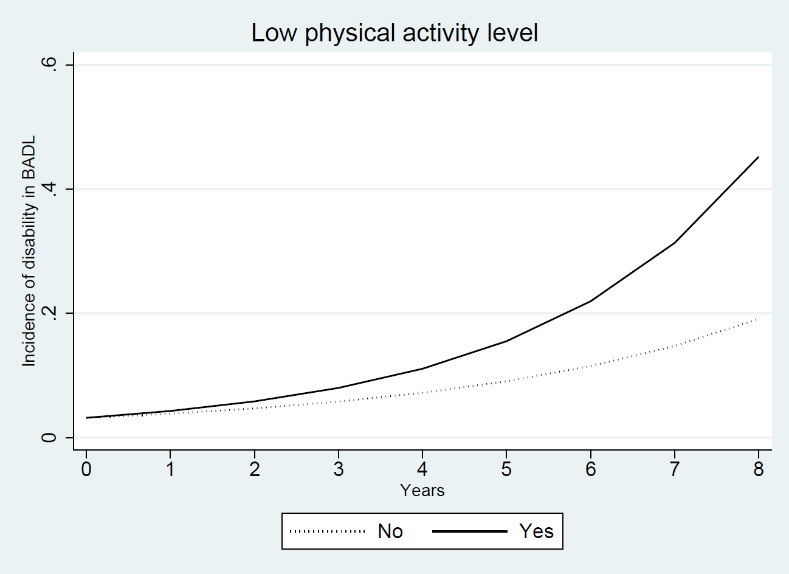

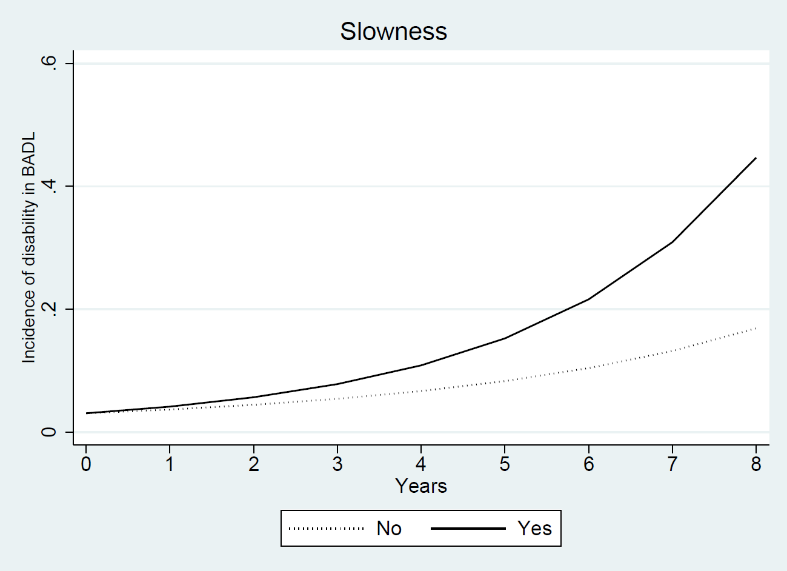


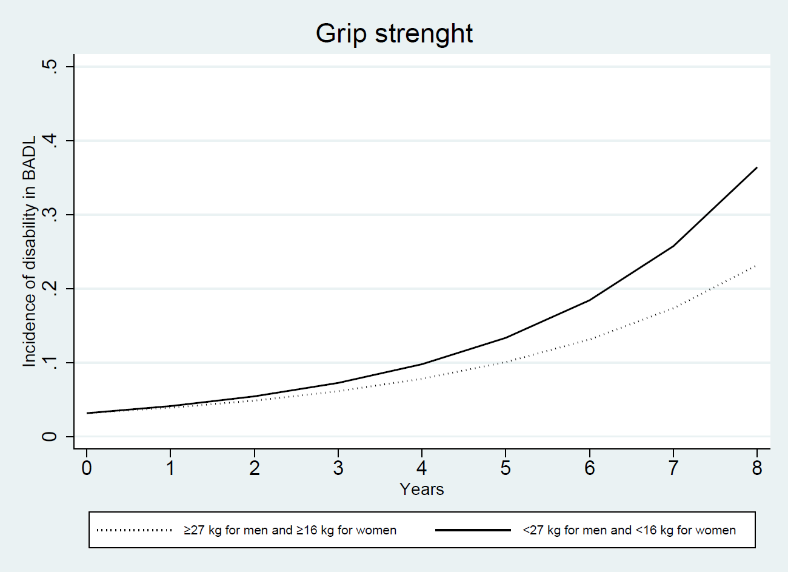


**Supplementary Figure 2.** Incidence of disability on BADL according to individual components of sarcopenia construct over eight-year follow-up – *English Longitudinal Study of Ageing* 2004/2005 – 2012/2013. Model adjusted by age, sex, race, marital status, schooling, total family wealth, smoking, alcohol intake, physical activity, systemic arterial hypertension, diabetes mellitus, cancer, lung disease, heart disease, stroke, osteoarthritis, osteoporosis, falls, depressive symptoms, memory score and BMI.


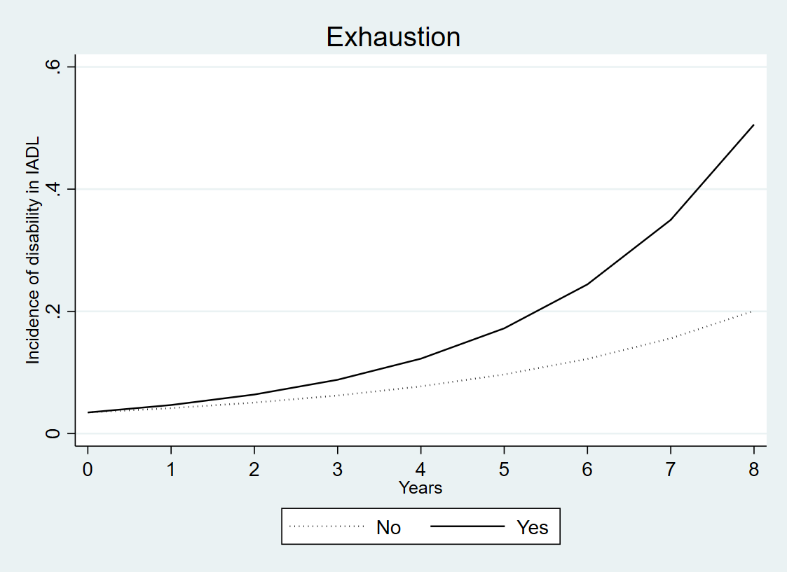

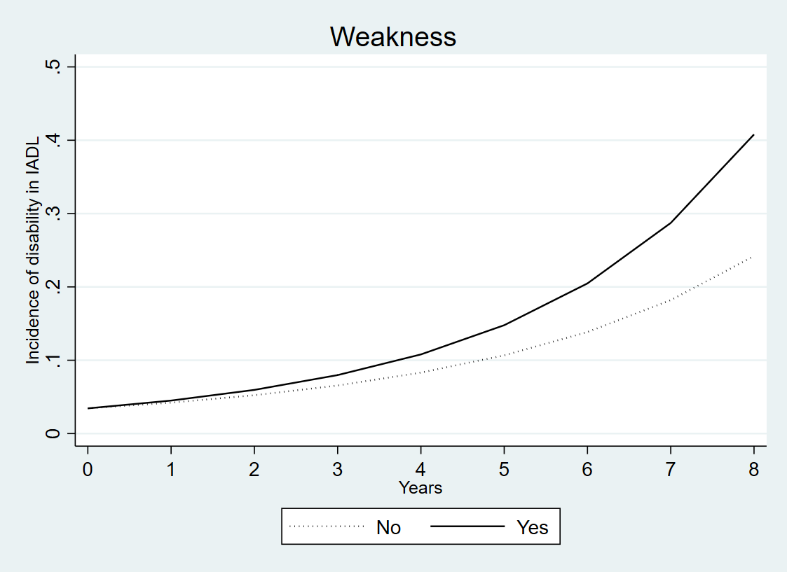


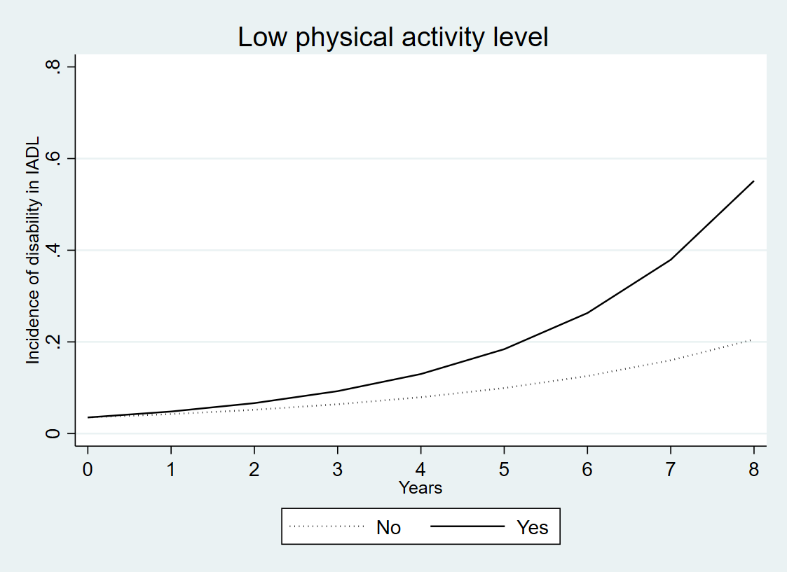

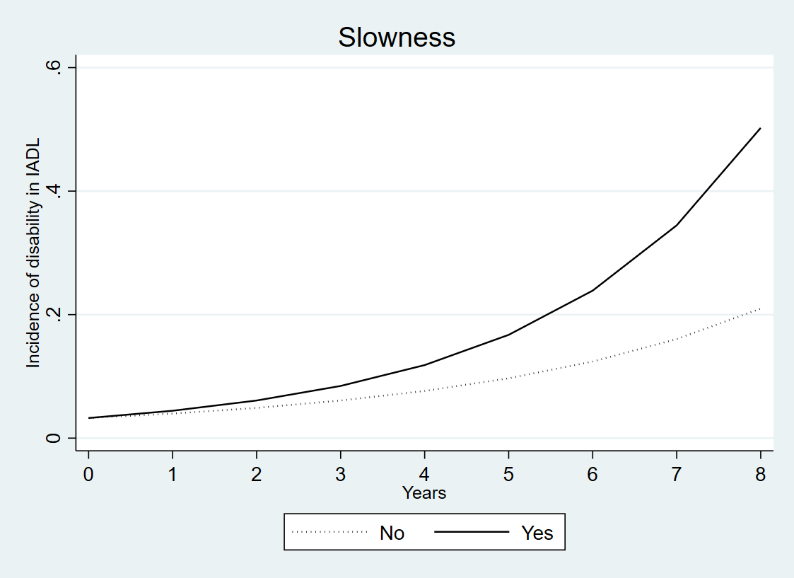


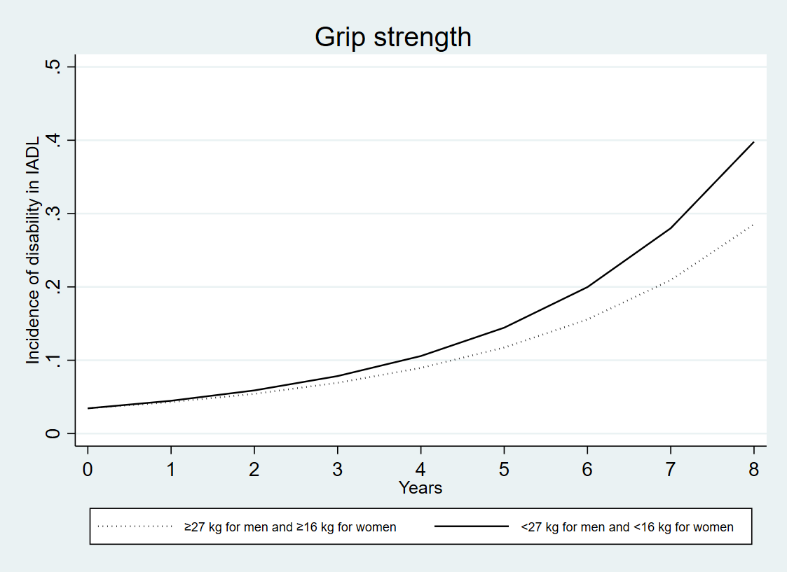


**Supplementary Figure 3.** Incidence of disability on IADL according to individual components of sarcopenia construct over eight-year follow-up – English Longitudinal Study of Ageing 2004/2005 – 2012/2013. Model adjusted by age, sex, race, marital status, schooling, total family wealth, smoking, alcohol intake, physical activity, systemic arterial hypertension, diabetes mellitus, cancer, lung disease, heart disease, stroke, osteoarthritis, osteoporosis, falls, depressive symptoms, memory score and BMI.

1. **Supplementary references**
2. Fried LP, Tangen CM, Walston J et.al. Frailty in Older Adults: Evidence for a Phenotype. J. Gerontol A Biol Sci Med Sci 2001;56(3):146–156.
3. Organization WH. Obesity: Preventing and Managing the Global Epidemic. World Health Organization; 2000
4. Radloff LS. The CES-D Scale: A Self-Report Depression Scale for Research in the General Population. Appl Psychol Meas. 1977;1(3):385-401.
5. Craig R, Mindell J, Hirani V, Joint Health Surveys Unit (Great Britain), Great Britain, National Health Service, et al. Health survey for England 2008: physical activity and fitness. London: National Centre for Social Research with permission of The NHS Information Centre; 2009.
6. Scholes S, Coombs N, Pedisic Z et al. Age- and sex-specific criterion validity of the health survey for England Physical Activity and Sedentary Behavior Assessment Questionnaire as compared with accelerometry. Am J Epidemiol 2014;179(12):1493–502.
7. Banks J, Breeze E, Lessof C NJ. Retirement, health and relationships of the older population in England: the 2004 English Longitudinal Study of Ageing. London: Institute for Fiscal Studies; 2006.
8. Cruz-Jentoft AJ, Bahat G, Bauer J et al. Sarcopenia: revised European consensus on definition and diagnosis. Age Ageing 2019;48:16-31.
9. Al Snih S, Markides KS, Ottenbacher KJ, Raji MA. Hand grip strength and incident ADL disability in elderly Mexican Americans over a seven-year period. Aging Clin Exp Res 2004;16(6):481–486.
10. Bohannon RW, Magasi S. Identification of dynapenia in older adults through the use of grip strength t-scores. Muscle & nerve 2015;51(1):102–105.
11. Lee RC, Wang Z, Heo M, Ross R, Janssen I, Heymsfield SB. Total-body skeletal muscle mass: development and cross-validation of anthropometric prediction models. Am J Clin Nutr 2000;72(3):796–803.
12. Al-Gindan YY, Hankey C, Govan L, Gallagher D, Heymsfield SB, Lean MEJ. Derivation and validation of simple equations to predict total muscle mass from simple anthropometric and demographic data. Am J Clin Nutr 2014;100(4):1041–51.
13. Spexoto MCB, Ramirez PC, Maximo RO, Steptoe A, Oliveira C, Alexandre TS. European Working Group on Sarcopenia in Older People 2010 (EWGSOP1) and 2019 (EWGSOP2) criteria or slowness: which is the best predictor of mortality risk in older adults? Age and Ageing 2022;51:1–10.
14. Delmonico MJ, Harris TB, Lee JS et al. Alternative definitions of sarcopenia, lower extremity performance, and functional impairment with aging in older men and women. J Am Geriatr Soc 2007;55:769–74.
15. Coin A, Sarti S, Ruggiero E et al. Prevalence of sarcopenia based on different diagnostic criteria using DEXA and appendicular skeletal muscle mass reference values in an Italian population aged 20 to 80. J Am Med Dir Assoc 2013;14:507–512.
16. Guralnik JM, Simonsick EM, Ferrucci L et al. A short physical performance battery assessing lower extremity function: association with self-reported disability and prediction of mortality and nursing home admission. J Gerontol 1994; 49(2):M85-94.
17. Guralnik JM, Ferrucci L, Pieper CF et al. Lower extremity function and subsequent disability: consistency across studies, predictive models, and value of gait speed alone compared with the short physical performance battery. J Gerontol A Biol Sci Med Sci 2000;55(4):M221-31.
18. Katz S, Ford AB, Moskowitz RW, Jackson BA, Jaffe MW. Studies of illness in the aged: A standardized measure of biological and psychosocial function. JAMA. 1963;185:914-919.
19. Oliveira DC, Máximo RO, Ramírez PC et al. Is slowness a better discriminator of disability than frailty in older adults? J Cachexia Sarcopenia Muscle 2021;2069–2078.
20. Lawton MP, Brody EM. Assessment of older people: self-maintaining and instrumental activities of daily living. The Gerontologist 1969;9(3):179-186.
21. Alexandre TS, Scholes S, Ferreira Santos JL, Duarte YAO, Oliveira C. The combination of dynapenia and abdominal obesity as a risk factor for worse trajectories of IADL disability among older adults. Clin Nutr 2018; 37(6 Pt A):2045–2053.
22. Banks J, Kumari M, Smith JP, Zaninotto P. What explains the American disadvantage in health compared with the English? The case of diabetes. JECH 2012;66(3):259–64.
23. Gallagher D, Kiss A, Lanctot K, Herrmann N. Depressive symptoms and cognitive decline: a longitudinal analysis of potentially modifiable risk factors in community dwelling older adults. J Affect Disord 2016;190: 235–240.
24. Huppert FA et.al. Cognitive function. In Banks J, Breeze E, Lessof C, Nazroo J, eds. Retirement, Health and Relationships of the Older Population in England: The 2004 English Longitudinal Study of Ageing, 217–242, 2006.
